# Examination of Genomic and Transcriptomic Alterations in a Morphologically Stable Line, MU1, Generated by Intergeneric Pollination

**DOI:** 10.3390/genes11020199

**Published:** 2020-02-15

**Authors:** Wei-Long Meng, Meng-Jie Zhao, Xiang-Bo Yang, An-Xing Zhang, Ning-Ning Wang, Zhao-Shi Xu, Jian Ma

**Affiliations:** 1 College of Agronomy, Jilin Agricultural University, Changchun 130118, China; mengweilongosj@163.com (W.-L.M.); zhanganxingosj@163.com (A.-X.Z.); yulanfang2020@sina.com (N.-N.W.); 2 Key Laboratory of Biology and Genetic Improvement of Triticeae Crops, Ministry of Agriculture, Institute of Crop Science, Chinese Academy of Agricultural Sciences (CAAS)/National Key Facility for Crop Gene Resources and Genetic Improvement, Beijing 100081, China; zhao_mengjie0815@163.com; 3 College of Agronomy, Jilin Agricultural Science and Technology University, Jilin 132101, China; winter0106j@163.com

**Keywords:** rice, intergeneric pollination, genetic variation, hormone signal transduction pathway

## Abstract

Interspecific hybridization creates genetic variation useful for crop improvement. However, whether pollen from a different genus affects the genomic stability and/or transcriptome of the recipient species during intergeneric pollination has not been investigated. Here, we crossed japonica rice cv. Z12 with the maize accession B73 (pollen donor) and obtained a morphologically stable line, MU1, exhibiting moderate dwarfism, higher tiller number, and increased grain weight compared with Z12. To reveal the genetic basis of these morphological changes in MU1, we performed whole-genome resequencing of MU1 and Z12. Compared with Z12, MU1 showed 107,250 single nucleotide polymorphisms (SNPs) and 23,278 insertion/deletions (InDels). Additionally, 5’-upstream regulatory regions (5’UTRs) of 429 and 309 differentially expressed genes (DEGs) in MU1 contained SNPs and InDels, respectively, suggesting that a subset of these DEGs account for the variation in 5’UTRs. Transcriptome analysis revealed 2190 DEGs in MU1 compared with Z12. Genes up-regulated in MU1 were mainly involved in photosynthesis, generation of precursor metabolites, and energy and cellular biosynthetic processes; whereas those down-regulated in MU1 were involved in plant hormone signal transduction pathway and response to stimuli and stress processes. Quantitative PCR (qPCR) further identified the expression levels of the up- or down-regulated gene in plant hormone signal transduction pathway. The expression level changes of plant hormone signal transduction pathway may be significant for plant growth and development. These findings suggest that mutations caused by intergeneric pollination could be the important reason for changes of MU1 in agronomic traits.

## 1. Introduction

Hybridization between genetically distinct plant species is an important evolutionary force. A possible explanation for the hybridization-associated genetic instability is that pollination of distant and incompatible species may cause biological stress to the recipient parent in diverse ways [1]. For instance, alien pollen release phytohormones and various compounds such as nitric oxide and reactive oxygen species [2], which could enter the stigma cells of the recipient parent during physical contact. This process may lead to abnormal physiological and biochemical changes and restrict the cellular machinery, eventually causing genomic instability.

Compared with interspecific hybridization, intergeneric pollination is more difficult to accomplish because the lack of homology between chromosomes of the two parental genotypes causes irregular meiosis [3]. However, intergeneric hybrids can be obtained using polyploidization or embryo rescue methods. Nonetheless, intergeneric hybrids and their progenies show various genomic, epigenomic, and transcriptomic differences compared with the parental genotypes [3,4,5,6,7]. Previous studies on rice-Zizania introgression lines showed that intergeneric pollination can also induce extensive genomic variation, based on molecular marker analysis and deep-coverage whole-genome resequencing [1,6,7,8]. Wang et al. identified 41,724 and 17,839 genome-wide homozygous single nucleotide polymorphisms (SNPs) and insertion/deletions (InDels) in a typical rice-Zizania introgression line RZ35, respectively. A nonsynonymous SNP in Pid3/Pi25, a blast resistance gene, which might improve the blast resistance of RZ35 [8]. These changes were mainly manifested in protein-coding genes and transposable element (TE)-related DNA segments [6]. Because of parapatric and sympatric distributions and shared pollinators of congeneric species, interspecific and intergeneric pollinations are a frequent occurrence in nature [9,10]. With exception of bona fide hybridization, alien pollen germinating on the stigma may cause biological stress to the recipient genome, resulting in genetic instabilities, similar with those caused by pathogen attack [1,11,12,13]. Genetic variation due to alien pollen may affect the evolution of plant genomes, in contrast to hybridization between intergeneric species.

Pollination by pollen from an incompatible species, especially a species belonging to a different genus, could generate homoploids or polyploids and lead to the introgression of genomic regions from one species to another [14,15,16,17]. Many studies show that interspecific hybridization causes extensive genomic rearrangements, single nucleotide polymorphisms (SNPs), and insertions/deletions (InDels) in the derived hybrids [18,19,20,21,22,23]. This phenomenon does not only occur in intergeneric pollinations, but also in interspecific hybrids. For example, in the introgression lines of Brassica napus, generated by interspecific hybridization between *Brassica napus* and *Brassica rapa*. a wide range of genomic alterations was detected, some of which were associated with alterations in yield-related traits [24]. Hybridization could also cause massive activation of silent TEs, thus acting as a “genome shock” [25]. For instance, hybridization between cultivated rice (*Oryza sativa*) and African rice (*Oryza glaberrima*) resulted in the transcriptional activation of the TE mPing [26]. A similar phenomenon was also observed in the introgression lines RZ35 developed from the hybridization between japonica rice cv. Matsumae and *Zizania latifolia*, where several TEs were transcriptionally activated or mobilized [8]. Extensive genomic changes could be the most fundamental reason, which enhanced the resistance to blast fungus [8]. 

Several mutation breeding methods have been developed for generating new cultivars in crop breeding programs. However, the application of mutations induced by intergeneric pollination has been seldom reported. In the present study, a morphologically stable line MU1 was generated by pollinating japonica rice cv. Z12 with the pollen of maize accession B73 using the “repeated pollination” approach. Line MU1 showed dwarfism and increased grain yield; both these traits are valuable for rice breeding. To investigate the genomic and transcriptomic effects of alien pollen on MU1, we performed whole-genome resequencing and genome-wide transcriptomic analyses of MU1 and Z12.

## 2. Materials and Methods

### 2.1. Plant Materials

**Figure 1:**
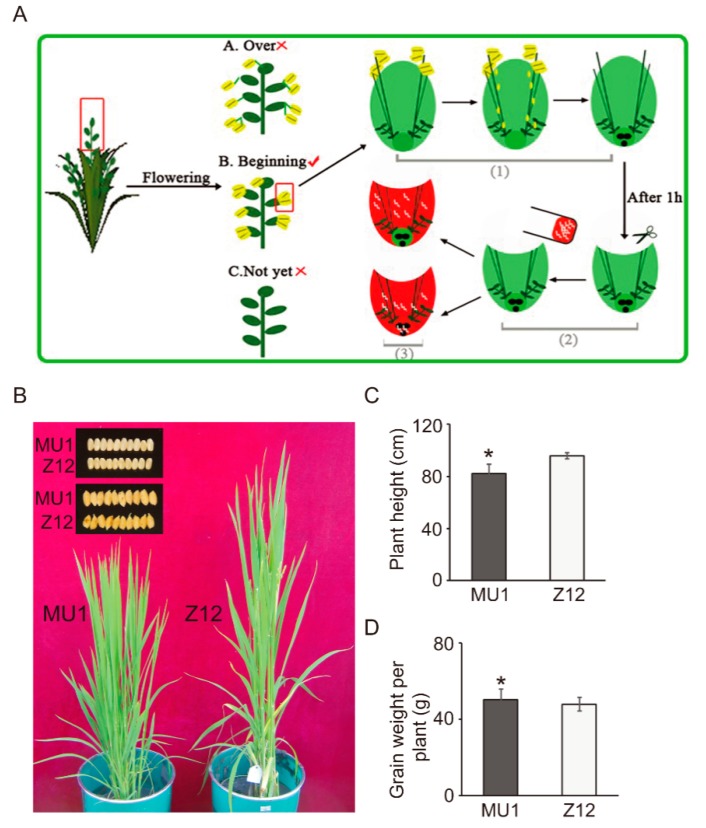
Diagrammatic illustration of the repeated pollination approach and analysis of phenotypic traits of the a morphologically stable line MU1. (**A**) Repeated pollination approach [28] used to generate a morphologically stable line. 1: Remove anthers; 2: Cut half of each flowering glume; 3: Intergenic pollination. (**B**) MU1 and Z12 (wild type) plants at the tillering stage and their seeds with/without husks. (**C**) Plant height of MU1 and Z12 plants at the heading stage. (**D**) Grain weight per plant. Plants of MU1 and Z12 were grown in the greenhouse. Data represent mean ± standard deviation (SD) of three biological replicates (n = 3), each comprising 20 plants. Significant differences between MU1 and Z12 are indicated with an asterisk (* *p* < 0.05; Student’s *t*-test).

MU1 rice lines were generated by pollinating japonica rice cv. Z12 with pollen from the maize accession B73 using the repeated pollination approach [27], with minor modifications, as shown in Figure 1A. To obtain stable inherited MU1, the resulting plants were self-pollinated for six generations (S6), and in each generation, only individuals showing dwarfism and constant or increased grain yield per plant were selected for reproduction. MU1, a stable S6 RIL, displaying dwarfism and increased grain yield per plant was selected for further analysis. MU1 and Z12 plants were grown in field trials at Jilin Agricultural University, Changchun, China for three consecutive years (2015–2017), with three replications each year. Values of agronomic traits represent the averages of measurements over 3 years, each with three replications (Table 1).

### 2.2. Genomic DNA Isolation and Whole-Genome Rresequencing

MU1 and Z12 plants are grown at a constant temperature of 28 °C and genomic DNA was extracted from the 7th expanded leaf of plants at the eight-leaf stage using the modified cetyl trimethyl ammonium bromide (CTAB) method and purified by phenol extraction. Three biological replicates of genomic DNA of each sample were used for whole-genome resequencing. Library construction and HiSeq2000 sequencing (Illumina) were carried out using standard protocols. Reads ≥90 bp in length were evaluated. Raw reads of Z12 were mapped onto the Nipponbare reference genome (MSU7.0 http://rice.plantbiology.msu.edu/index.shtml) using the Burrows-Wheeler Aligner with the parameter mem-t4-k32-M. Reads showing unique mapping to the reference genome and mapping quality score > 30 (Phred scale) were used for genome assembly. Perl scripts were used to produce a theoretical Z12 reference genome including the sequences that contained nucleotide variation compared with Nipponbare. Next, the reads of MU1 were aligned to the theoretical Z12 genome to discover SNPs and InDels using SAMtools. To minimize the false-positive rates of SNPs and InDels, reads with Phred score ≥30 and coverage ranging from ≥10 and ≤100 were selected.

### 2.3. Distribution of Nucleotide Polymorphisms and Identification of Mutant Genes

To illustrate the pattern of genome-wide nucleotide variations and identify hypermutation regions (HMRs) in the MU1 genome, a sliding window at 100-kb intervals was employed to estimate polymorphisms in each chromosome using Perl scripts [28]. Figures were generated using the ggplot2 package in R (https://cran.r-project.org/web/packages/ggplot2). The locations of HMRs in MU1 were compared with the domestication-related regions of rice identified previously [29]. To assess the base substitution rates, the Ts/Tv ratio was calculated for each chromosome in MU1. Based on the Generic Feature Format Version 3 (GFF3) files of the annotated Nipponbare reference genome, SNPs were annotated as genic or intergenic. The proportion of genic SNPs in CDSs, UTRs, and introns was also calculated. Furthermore, SNPs in CDSs were categorized as synonymous and nonsynonymous, depending on the predicted amino acid substitutions. The nonsynonymous and large-effect SNPs were further analyzed in the Gene Ontology (GO) database using the AgriGO analysis tool [30].

### 2.4. RNA Isolation and Transcriptome Sequencing

The shoots of MU1 and Z12 plants were harvested for RNA-seq and total RNA was isolated using the Trizol Reagent (Transgen), according to the manufacturer’s protocol. RNA-seq libraries were constructed for shoots and sequenced with HiSeq2000 (Illumina), according to standard protocols (Illumina). Raw sequence reads were cleaned by removing adaptor sequences and low-quality reads using Fastx-tools (http://hannonlab.cshl.edu/fastx_toolkit). Reads were mapped to the Oryza sativa genome (MSU Release 7.0) using Tophat [31]. The gene was identified as DEGs if|log2foldchang|>1 (*p*_adjusted < 0.05). Differential expression analysis for sequence count data. Genome Biol. (DESeq)]. Differential gene expression analysis was performed using Cuffdiff, as described previously [32]. 

### 2.5. RNA Extraction and Gene Expression Analysis by Quantitative PCR (qPCR)

Total RNA was extracted using Trizol reagent according to the protocol (TIANGEN, Beijing, China) and then treated by DNase I. PrimeScript First-Strand cDNA Synthesis Kit (TaKaRa, Kusatsu, Japan) was used to synthesized cDNA of the total RNA. qPCR was used to analyze the relative expression levels of several DEGs. qPCR was performed on the ABI Step One Plus qPCR System (Applied Biosystems, Foster City, CA, USA) using SYBR green dye (Applied Biosystems, Foster City, CA, USA) and gene-specific primers (Table S9). The glyceraldehyde-3-phosphate dehydrogenase (GAPDH) gene, a housekeeping gene, was used as the internal control gene for data normalization [33]. Three biological repetitions and three technical repetitions were performed. The data was shown as the means ± SD of all the replicates.

## 3. Results

### 3.1. Phenotypic Characterization of MU1

We generated a series of mutant rice lines using a sexual hybridization approach, previously known as repeated pollination [27], as shown in Figure 1A. Approximately 6000 M0 seeds were generated and matured from more than 15,000 pollination events. Through field investigations, 226 dwarf plants relative to Z12 were selected from the M1 population derived from 44 M0 seeds. There are about 6% MU1-looking plants in total “M1” generation. Each dwarf M1 plant was self-pollinated for more than five generations, and progenies with a dwarf phenotype and an increased or unchanged individual yield relative to Z12 were selected in each generation for selfing. Among the resulting recombinant inbred lines (RILs), a stable line, MU1, was identified. Compared with Z12, MU1 not only displayed short height and increased individual yield (Figure 1B–D, Table 1) but also showed increased tiller number and 1000-grain weight and reduced germination rate (Table 1). These results indicate that intergeneric pollination with maize, whose pollen are huge and easily accessible, is an effective way to induce multiple phenotypic changes in rice that could be used to develop new germplasm.

### 3.2. Analysis of Genomic Variation in MU1

To determine the effect of pollination by an intergeneric species on genome stability, we conducted whole-genome resequencing of MU1 and Z12. A total of 89.4 million reads of Z12 and MU1 were mapped onto the Nipponbare reference genome (MSU7.0) (Table S1). The uniquely-mapped reads (>99% of the total mapped reads) showed 13.2× and 21.9× coverage of the Z12 and MU1 genomes, respectively (Table S1). Alignment of MU1 reads against the Z12 reference genome (as described in Materials and Methods) revealed a total of 107,250 SNPs and 40,332 InDels in MU1 (Table S2). There are 30.3 SNPs and 10.8 InDels per 100 Kb sliding window on average (Table S2). 

Interspecific and intergeneric pollination could lead to the introgression of uncharacterized DNA segments from the donor species into the recipient genome; this characteristic has been widely used in crop breeding for introducing useful traits [7,13,33]. To investigate whether maize genomic DNA fragments were introgressed into the MU1 genome, clean reads of Z12 and MU1 obtained by resequencing were aligned with the maize reference genome (B73 RefGen_v3). A small proportion (7.8%) of the clean MU1 reads mapped to the maize genome (Table S3), and all of these reads also matched the rice genome. Reads matching both maize and rice genomes probably represent genomic fragments conserved between the two plant species, and not the introgression of maize genomic DNA fragments into the MU1 genome.

### 3.3. Classification of SNPs in MU1

**Figure 2:**
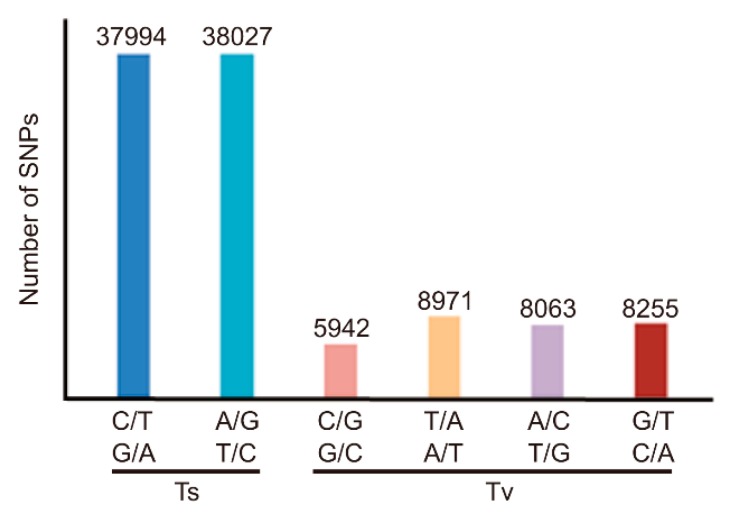
Comparisons of transitions (Ts) and transversions (Tv) in MU1 and Z12.

**Figure 3:**
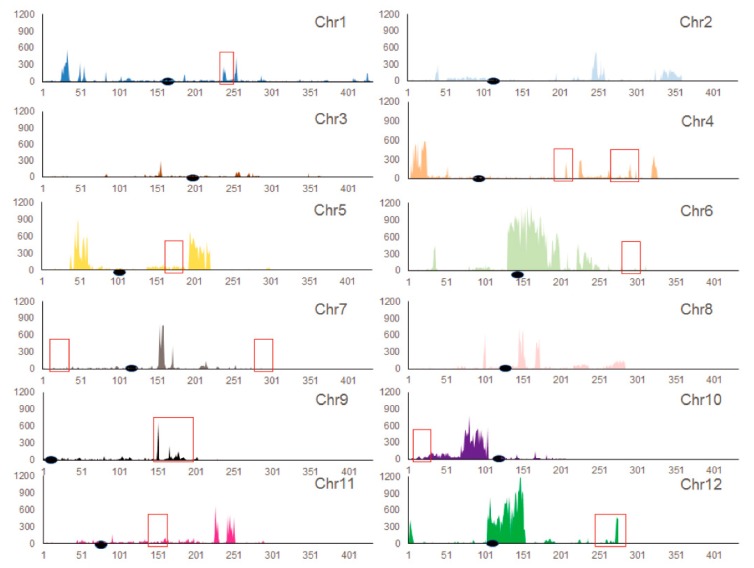
Distribution of SNPs within 100-kb windows across each chromosome. Different rice chromosomes (Chr-12) are indicated in different colors. The X-axis represents the length of each chromosome divided by 0.1 Mb. The Y-axis indicates the number of SNPs. Black solid ellipse represents the centromere. Red frames indicate the domestication-related regions identified in japonica rice [28].

Analyses of the global patterns of genetic variation in MU1 revealed that G/A and C/T transitions (Ts) were the two most common types of substitutions, leading to a higher rate of Ts than transversions (Tv; Ts/Tv ratio = 2.38) (Figure 2, Table S4). Additionally, several HMRs were identified in the genome of MU1, which were specific to MU1. Most of these HMRs occurred in the euchromatic regions of each chromosome, except chromosomes 6 and 12, where HMRs were located near the centromere (heterochromatic region) (Figure 3). Five of these HMRs were associated with domestication-related regions of rice reported previously (Figure 3) [27]. Thus, intergeneric pollination triggered extensive genomic changes in MU1.

**Figure 4:**
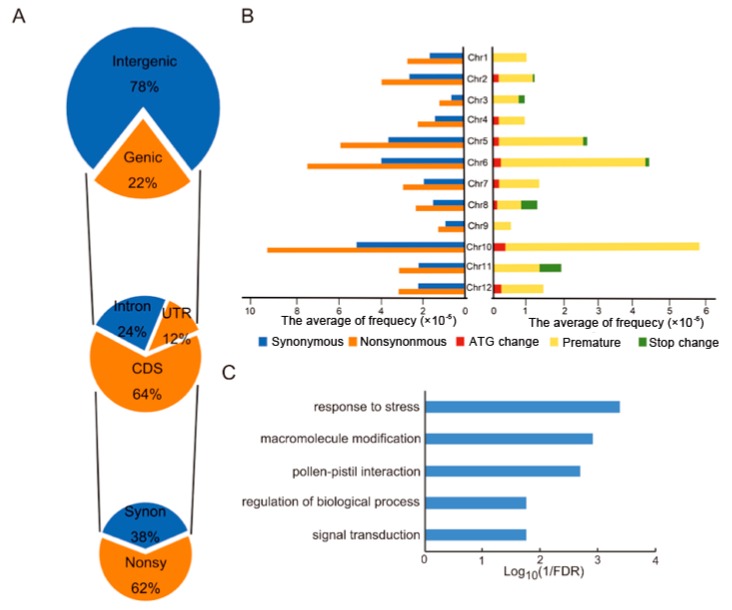
Annotation of homozygous SNPs identified in MU1 based on the comparison with Z12. (**A**) Number and percentile of homozygous SNPs located in genic and intergenic regions. SNPs located in genic regions were further annotated. (**B**) Average frequency distributions of synonymous, nonsynonymous, and large-effect SNPs on each of the 12 rice chromosomes. The X-axis represents frequency distributions, whereas the Y-axis represents the different chromosomes. (**C**) Gene Ontology (GO) enrichment analysis of genes containing nonsynonymous and large-effect SNPs.

The majority of SNPs in MU1 were located in intergenic regions (78%), while only 22% of SNPs mapped to genic regions (Figure 4A). 14,952 (64%), 2880 (12%), and 5455 (24%) of all the genic SNPs were located in coding sequences (CDSs), UTRs, and introns, respectively. Among the SNPs located in CDSs, 62% were nonsynonymous, and 38% were synonymous (Figure 4A). Additionally, 6662 genes contained more than one nonsynonymous SNP (Table S5). The average frequency of synonymous and nonsynonymous SNPs varied significantly among the 12 rice chromosomes (Figure 4B). For instance, SNPs appear most frequently on chromosome 10. Most of them occurred in the premature region, a few SNPs cause ATG changes, and no SNP causes stop change, most of which resulted in non-synonymous mutations in Chromosomes 10. All the SNPs are distributed in premature of CDS of Chromosomes 1 and 9, none distribute in ATG or stop region. Chromosomes 5, 6, and 10 contained more synonymous and nonsynonymous CDS SNPs than the other nine chromosomes (Figure 4B). Similarly, the distribution of large-effect SNPs, which result in premature stop codons, loss of ATG start sites, and replacement of nonsense mutations by sense codons (Stop change), was non-random among the rice chromosomes, with enrichment in chromosomes 5, 6, and 10 (Figure 4B). Gene Ontology (GO) annotations revealed that genes containing nonsynonymous or large-effect SNPs in their CDSs were classified into various categories, such as response to stress (false discovery rate [FDR] = 4.1 × 10^−4^), protein modification (FDR = 1.2 × 10^−3^), pollen-pistil interaction (FDR = 2 × 10^−3^), regulation of biological process (FDR = 1.7 × 10^−2^), and signal transduction (FDR = 1.7 × 10^−2^), suggesting that these amino acid substitutions may lead to novel phenotypes in MU1 (Figure 4C).

**Figure 5:**
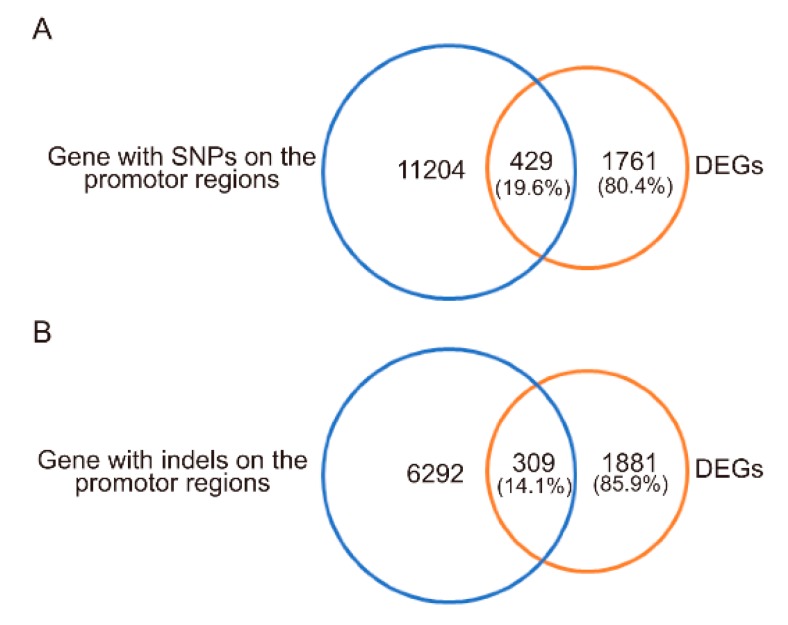
Number of differentially expressed genes (DEGs) carrying nucleotide polymorphisms in their putative promotor regions in MU1. (**A**) Number of DEGs with SNPs in promotor regions. (**B**) Number of DEGs with InDels in promotor regions.

To investigate whether the transcriptomic changes in MU1 were associated with genomic variation, all SNPs and InDels in the putative promoter regions (2 kb upstream of the transcription start site) were identified. The results revealed that 429 (19.6%) and 309 (14.1%) differentially expressed genes (DEGs) contained at least one SNP or InDel, respectively, in their promoter regions (Figure 5). There are so many cis-acting elements that could be bound by transcription factors in the promoter region, which could regulate the gene expression level. SNPs or InDels in the promoter regions could be responsible for changes of DEGs in MU1.

### 3.4. Changes in GO Annotations of Genes Differentially Expressed between MU1 and Z12

**Figure 6:**
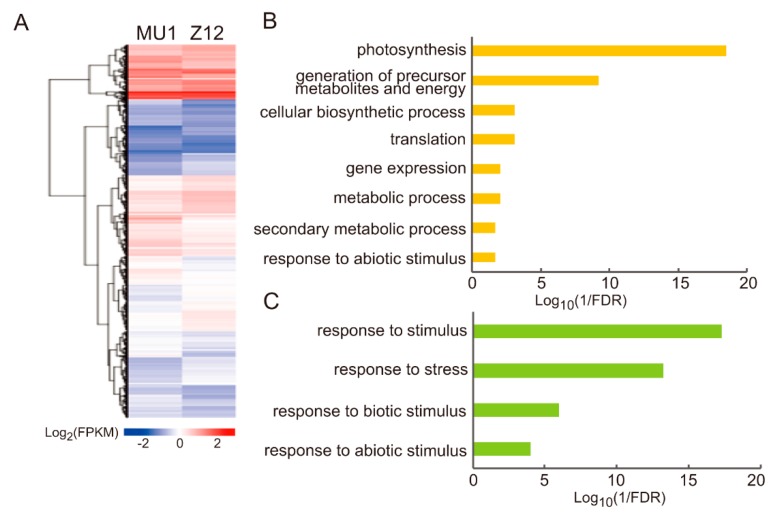
Genome-wide transcriptomic differences between MU1 and Z12. (**A**) Heatmap of genes differentially expressed between MU1 and Z12. Biological function of up-regulated genes. (**B**) And down-regulated genes (**C**) in MU1, based on GO analysis.

To understand the potential effects of pollination by an alien species on gene expression in the host species, we compared the gene expression in MU1 relative to that in Z12. RNA extracted from the leaves of MU1 and Z12 were used to construct RNA-seq libraries with three biological replicates. Using stringent filtering criteria (see Materials and Methods for details), 2336 DEGs showing significant differences in expression relative to Z12 were identified in MU1 (Figure 6A, Table S6). Among these genes, 1078 were up-regulated, and 1258 were down-regulated in MU1. Hierarchical clustering analysis revealed that the transcriptional profile was largely altered in MU1 compared with Z12 (Figure 6A). GO enrichment analysis showed that the up-regulated genes were enriched in several distinct biological categories, including photosynthesis (FDR = 3.20 × 10^−19^), generation of precursor metabolites and energy (FDR = 5.80 × 10^−10^), cellular biosynthetic process (FDR = 8 × 10^−4^), macromolecule biosynthetic process (FDR = 8 × 10^−4^), and translation (FDR = 8 × 10^−4^) (Figure 6B, Table S7), while the down-regulated genes were stratified into multiple stress/stimulus response-related processes (Figure 6C, Table S8). 

### 3.5. Changes in Signal Transduction Pathways in MU1 

**Figure 7:**
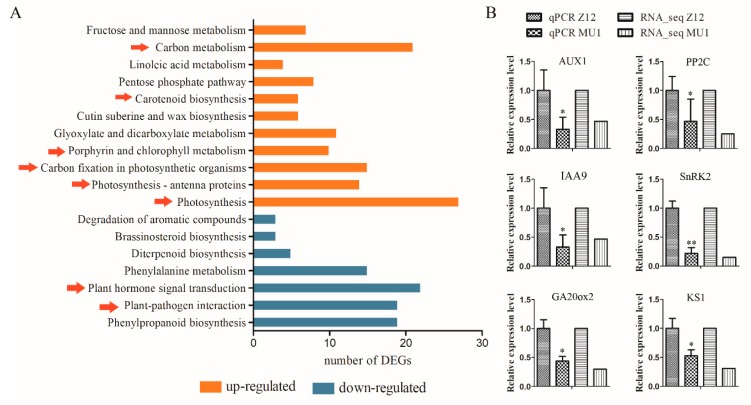
Kyoto Encyclopedia of Genes and Genomes (KEGG) pathway analysis and qPCR of genes differentially expressed in MU1 compared with Z12. (**A**) KEGG enrichment analysis. The Y-axis represents various pathways. The X-axis represents the numbers of DEGs. Up-regulated pathways are indicated in orange, and down-regulated pathways are indicated in blue (*p* < 0.05). (**B**) Expression analysis of DEGs involved in the plant hormone signal pathway. The relative expression level of some DEGs were determined, such as auxin transporter-like protein 1 (AUX1, Os09g0490200), IAA9 (Os02g0805100), protein phosphatase 2C (PP2C, Os09g0325700), SNF1-related kinase2 (SnRK2, Os03g0610900), Gibberellin20 oxidase 2 (GA20ox2, Os01g0883800), and GA biosynthesis gene KS1 (Os04g0611800).

To explore the effect of alien pollen on signal transduction pathways, we analyzed the DEGs in the Kyoto Encyclopedia of Genes and Genomes (KEGG) database [34]. A total of 18 signal pathways displayed significant differences between MU1 and Z12 (*p* < 0.05). Nine pathways, including photosynthesis, photosynthesis-antenna proteins, carbon fixation in photosynthetic organisms, porphyrin and chlorophyll metabolism, glyoxylate and dicarboxylate metabolism, cutin, suberine and wax biosynthesis, carotenoid biosynthesis, pentose phosphate pathway, linoleic acid metabolism, carbon metabolism, and fructose and mannose metabolism, showed an upward adjustment (Figure 7A). Most of these pathways are related to anabolic processes in plants. DEGs involved in plant hormone signal transduction pathway showed the most abundant number of enrichments in down-regulated pathways. A previous study showed that plant hormone regulation is independent of plant growth and development [35]. Additionally, plant-pathogen interaction was decreased in MU1. Together, these results suggest that dwarfism and increased tillers in line MU1 resulted from the changes in the expression of genes involved in various signaling transduction pathways [36,37,38]. 

qPCR was conducted to verify the expression level of DEGs in MU1 and Z12. Total RNA was extracted from the leaves of MU1 and Z12 plants and used to analyze the expression levels of auxin transporter-like protein 1 (AUX1, OS09G0490200), IAA9 (OS02G0805100), protein phosphatase 2C (PP2C, OS09G0325700), SNF1-related kinase2 (SnRK2, OS03G0610900), Gibberellin20 oxidase 2 (GA20ox2, Os01g0883800), and GA biosynthesis gene KS1 (Os04g0611800) by qPCR [36,37,38] (Figure 7B). All these genes were significantly down-regulated in MU1 compared with Z12. 

### 3.6. Verification of RNA-seq Data

**Figure 8:**
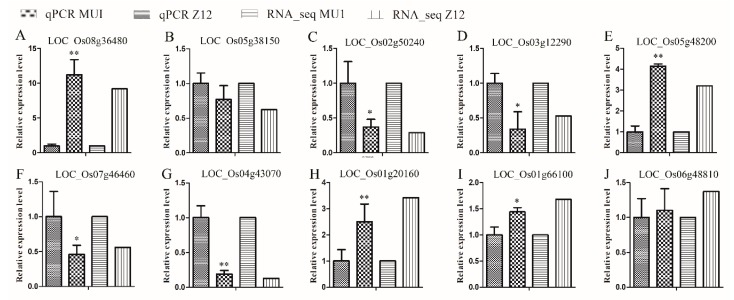
Transcriptomic changes in MU1 compared with Z12. Data represent mean ± SD of three biological replicates. Asterisks indicate significant differences between MU1 and Z12 (* *p* < 0.05; ** *p* < 0.01; Student’s *t*-test).

To verify the authenticity of RNA-seq analysis and generate supplementary data, 10 DEGs (LOC_Os08g36480, LOC_Os05g38150, LOC_Os02g50240, LOC_Os03g12290, LOC_Os05g48200, LOC_Os07g46460, LOC_Os04g43070, LOC_Os06g48810, LOC_Os01g66100, and LOC_Os03g58040) including five up-regulated and five down-regulated genes were randomly selected for expression analysis by qPCR. Primers used for qPCR are listed in supplementary Table S9. The results of qPCR analysis were consistent with those of RNA-seq analysis (Figure 8). This confirmed the reliability of our RNA-seq data. 

## 4. Discussion

Numerous studies have shown that interspecific hybridization can lead to genome-wide variation in the resulting progeny [19,39,40,41,42,43,44,45]. In this study, based on whole-genome resequencing and RNA-seq analysis, we showed that intergeneric pollination of japonica rice cv. Z12 with maize pollen induced genome-wide nucleotide variation and transcriptomic changes in the resulting a morphologically stable line, MU1. The quantity of both SNPs and InDels in MU1 was much higher than that in the rice-Zizania introgression line. Additionally, the frequency of Ts was significantly higher than that of Tv in MU1. This nucleotide substitution bias is similar with the rice-Zizania line [8] and is broadly consistent with previous studies on natural species [30,46,47], thus corroborating that genomic variation in MU1 was triggered by intergeneric pollination. A small amount of Zizania-specific DNA was found in the rice-Zizania introgression lines [7]. However, we found no introgression of maize genomic DNA fragments in MU1, indicating that the phenotypic changes of MU1 were generated de novo, resulting from genomic changes provoked by intergeneric pollination.

In the present study, nucleotide variation in MU1 tended to localize in clusters on specific chromosomes rather than being randomly distributed across different chromosomes or along the length of a specific chromosome. Although the non-random positioning of SNPs has been reported previously in rice cultivars [48,49,50], we note that only a few of the HMRs found in MU1 were shared among the present-day rice cultivars, indicating that new genetic variation was induced in rice by intergeneric pollination with maize. Nucleotide substitution rates are known to differ across the genomes of natural plant species because of different degrees of selection or genetic drift against mutations [51,52,53]. Some studies have suggested that nucleotide substitution rates might be accelerated in chromosomal regions under natural selection or determined by genetic drift in neutral regions [54]. Therefore, the genomes of natural species are chimeras of discontinuous segments, which possess unique histories and contribute differentially to the evolution of a species. Our study provides a new example in support of this hypothesis because most HMRs were identified across each of the 12 chromosomes of MU1. 

Transcriptome analysis uncovered the expression of many genes that were altered in MU1 compared with Z12 and were involved in multiple biologically important pathways. The amount of up-regulated genes in MU1 were mainly enriched in photosynthesis, generation of precursor metabolites and energy, cellular biosynthetic process as well as translation, all of which were suggested to contribute to anabolism in plants [55]. These induced pathways are likely responsible for the increase in tiller number and 1000-grain weight in MU1. The changes in plant hormones also caught our attention. Expression levels of among of genes involved in plant hormones transduction pathway were down-regulated in MU1 compared with Z12, such as ABA, GA, IAA, and ET signaling pathways. Previous studies showed that hormone levels in plants play important roles in regulating plant growth and development including tiller growth and plant height [51]. This change was mainly manifested in the reduction of transcript levels of genes involved in ABA, IAA, and ET signal pathways in MU1. Many genes were significantly down-regulated in these pathways, including AUX1, PP2C, IAA9, SnRK2, GA20ox2, and KS1. Liu et al. showed that IAA plays a critical role in inhibiting tiller bud growth [56]. Gibberellins (GAs) are a kind of independent hormone in plant development, which significantly affect plant height and yield [57]. Mutants defective in GA metabolic enzyme displayed a dwarf phenotype, which could be restored by the application of exogenous GA [58]. In this study, qPCR analysis showed the expression levels of GA biosynthesis genes such as OsGA20ox2 and OsKS1 were decreased (Figure 6C). Additionally, KEGG pathway analysis showed the biosynthesis of zeatin, a type of cytokinin [59], was significantly increased in MU1. Previously, research in tobacco showed that a distinct reduction in apical dominance and increase in tillering stage was closely associated with the zeatin content of plants [60]. The dwarfism and increased tillers phenotypes observed in MU1 in this study may be caused by the changes in the expression level of genes involved in various hormone signaling transduction pathways. Moreover, the alignment of clean reads of MU1 and Z12 showed that only 7.8% of MU1 reads mapped to the maize genome, and these sequences also matched the rice genome. These results suggest that maize genomic DNA fragments may intensify rather than integrate into the rice genome via intergeneric pollination.

## 5. Conclusions

The results of whole-genome resequencing and RNA-seq analysis showed that intergeneric pollination with maize pollen induced genome-wide nucleotide variations as well as transcriptomic changes in rice, which were most likely responsible for the phenotypic changes in the morphologically stable line MU1. These genomic and phenotypic variations could be readily utilized for crop improvement.

## Figures and Tables

**Table 1:** Plant height, germination rate, panicle number, individual yield, and 1000-grain weight of MU1 and Z12 plants.

Line	Plant Height (cm)	Germination Rate (%)	No. of Effective Panicles	1000-Grain Weight (g)	No. of Grains Per Ear	Yield Per Acre (kg)
MU1	82.4 ± 6.8 **	81.2 ± 4.6 *	22.2 ± 3.8 **	22.22 ± 0.43 *	103.5 ± 6.4 **	560.63 ± 6.86 **
Z12	95.9 ± 2.2	86.3 ± 1.7	16.4 ± 2.4	21.35 ± 0.46	142.7 ± 5.6	532.42 ± 5.17

Plant height was determined at the ripening stage. * *p* < 0.05, ** *p* < 0.01 (Student’s *t*-test).

## Supplementary Materials

The following are available online at https://www.mdpi.com/2073-4425/11/2/199/s1. Table S1. Numbers of Reads and coverage of rice reference genome; Table S2. SNPs and indels in MU1 compared with Z12; Table S3. Comparison of Z12 and MU1 reads with maize reference genome; Table S4. Conversion and transversion ratio of SNPs in each chromosome of MU1; Table S5. List of nonsynonymous SNPs; Table S6. Differentially expressed genes in MU1 compared with Z12; Table S7. GO Enrichment of up-regulated genes in MU1; Table S8. GO Enrichment of down-regulated in MU1; Table S9. Primers used for real-time qRT-PCR.

## Abbreviations

qPCR	quantitative PCR
RILs	recombinant inbred lines
AUX	auxin transporter-like protein
PP2C	protein phosphatase 2C
GA	gibberellin acid
SnRK2	SNF1-related kinase2
DEG	differentially expressed genes
KEGG	Kyoto Encyclopedia of Genes and Genomes
